# Supplementary material for: The Role of Digital Technology in Curbing COVID-19
Source: Int J Environ Res Public Health. 2022 Jul 7;19(14):8287. doi: 10.3390/ijerph19148287 (PMC9320375; doi:10.3390/ijerph19148287)
Supplement: Supplementary file 1 [file ijerph-19-08287-s001.zip › ijerph-1756070-supplementary.pdf]

Online supplementary

Table S1. Summary of the digital health solutions terminology.

Terminology	Applications
**Telemedicine/digital medicine**	To provide online consultations for people requiring healthcare services and to support health care providers in decision-making [1].
**Telehealth/e-health /remote health care**	To enable remote monitoring of COVID-19 patients who are extremely vulnerable (i.e. diabetic patients and those with hypertension or COPD) during the pandemic [1].
**Video conferencing**	Mostly applied to primary care doctors and general practitioners for triage before coming to the clinic [2,3].
**Telerehabilitation/online sessions**	Online sessions to provide support for physical activity and mental health and well-being [4,5].
**Mobile health applications**	Tracking the symptoms and quarantining, booking appointments and vaccinations [6].
**Artificial intelligence**	Screening people in public places like airports to provide a quick diagnosis [7].
**Machine learning**	To predict the future of the pandemic, the number of cases, and the rate of mortality [8].
**Virtual reality**	To provide disease awareness and training for healthcare professionals, as well as physical rehabilitation for isolated COVID-19 patients [9].
**Internet of Things**	To reduce the spread of the infection by linking devices and mobile applications, location notifications, and social distance notifications [10].

Note: COPD, Chronic Obstructive Pulmonary Disease.

Figure S1. Examples from the literature of the digital technologies and applications used to mitigate COVID-19.

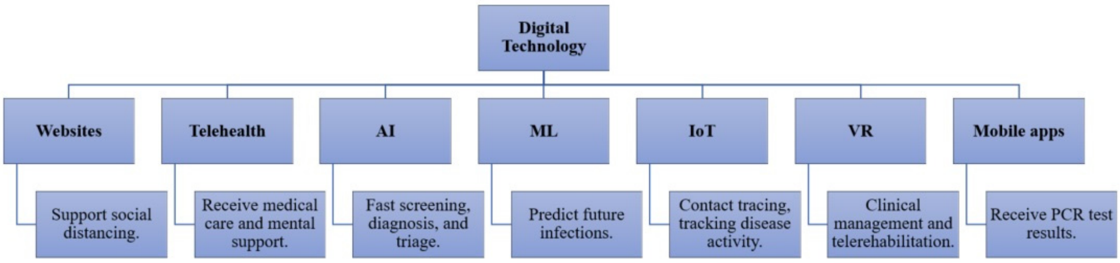

Note: AI, artificial intelligence; ML, machine learning; IoT, internet of things; VR, vertical reality; and PCR, polymerase chain reaction

Reference

1. Hollander, J. E.; Carr, B. G., Virtually perfect? Telemedicine for COVID-19. *New England Journal of Medicine* **2020,** 382, (18), 1679-1681.
2. Car, J.; Koh, G. C.-H.; Foong, P. S.; Wang, C. J., Video consultations in primary and specialist care during the covid-19 pandemic and beyond. *bmj* **2020,** 371.
3. Silsand, L.; Severinsen, G.-H.; Berntsen, G., Preservation of Person-Centered Care Through Videoconferencing for Patient Follow-up During the COVID-19 Pandemic: Case Study of a Multidisciplinary Care Team. *JMIR formative research* **2021,** 5, (3), e25220.
4. Adiukwu, F.; Orsolini, L.; Bytyçi, D. G.; El Hayek, S.; Gonzalez-Diaz, J. M.; Larnaout, A.; Grandinetti, P.; Nofal, M.; Pereira-Sanchez, V.; Da Costa, M. P., COVID-19 mental health care toolkit: an international collaborative effort by Early Career Psychiatrists section. *General Psychiatry* **2020,** 33, (5).
5. Zhou, X.; Snoswell, C. L.; Harding, L. E.; Bambling, M.; Edirippulige, S.; Bai, X.; Smith, A. C., The role of telehealth in reducing the mental health burden from COVID-19. *Telemedicine and e-Health* **2020,** 26, (4), 377-379.
6. Adeniyi, E. A.; Awotunde, J. B.; Ogundokun, R. O.; Kolawole, P. O.; Abiodun, M. K.; Adeniyi, A. A., Mobile health application and COVID-19: Opportunities and challenges. *Journal of Critical Reviews* **2020,** 7, (15), 3481-3488.
7. Vaishya, R.; Javaid, M.; Khan, I. H.; Haleem, A., Artificial Intelligence (AI) applications for COVID-19 pandemic. *Diabetes & Metabolic Syndrome: Clinical Research & Reviews* **2020,** 14, (4), 337-339.
8. Kushwaha, S.; Bahl, S.; Bagha, A. K.; Parmar, K. S.; Javaid, M.; Haleem, A.; Singh, R. P., Significant applications of machine learning for COVID-19 pandemic. *Journal of Industrial Integration and Management* **2020,** 5, (4).
9. Singh, R. P.; Javaid, M.; Kataria, R.; Tyagi, M.; Haleem, A.; Suman, R., Significant applications of virtual reality for COVID-19 pandemic. *Diabetes & Metabolic Syndrome: Clinical Research & Reviews* **2020,** 14, (4), 661-664.
10. Nasajpour, M.; Pouriyeh, S.; Parizi, R. M.; Dorodchi, M.; Valero, M.; Arabnia, H. R., Internet of Things for current COVID-19 and future pandemics: An exploratory study. *Journal of healthcare informatics research* **2020**, 1-40.
